# Efficacy and safety of Dysmenorrhea Patch acupoint application in women with primary dysmenorrhea: a randomized double-blind controlled trial

**DOI:** 10.3389/fendo.2026.1728199

**Published:** 2026-02-25

**Authors:** Yuan Gao, Ming Yang Li, Chu Ting Wu, Xiao Yan Dong, Jia Wei Hu, Yu Ran Li, Xiao Yun Zhang, Huan Gan Wu, Chun Yan Zhang

**Affiliations:** 1 Department of Acupuncture and Moxibustion, Yueyang Hospital of Integrated Traditional Chinese and Western Medicine, Shanghai University of Traditional Chinese Medicine, Shanghai, China; 2 Department of Ultrasound, Yueyang Hospital of Integrated Traditional Chinese and Western Medicine, Shanghai University of Traditional Chinese Medicine, Shanghai, China; 3 Department of Pathology, LongHua Hospital Shanghai University of Traditional Chinese Medicine, Shanghai, China; 4 Shanghai Research Institute of Acupuncture and Meridian, Shanghai, China

**Keywords:** acupoint application, dysmenorrhea patch, efficacy evaluation, primary dysmenorrhea, Qi stagnation and blood stasis, randomized double-blind controlled trial

## Abstract

**Background:**

Primary dysmenorrhea (PD) is defined as pain occurring with menses in the absence of pelvic pathology. Traditional Chinese Medicine offers various therapeutic approaches for PD management. Dysmenorrhea Patch, an acupoint application therapy, shows promise for PD management, but its clinical efficacy and safety require further evaluation. This study aimed to assess the efficacy and safety of the Dysmenorrhea Patch in PD.

**Methods:**

This multicenter, randomized, double-blind, placebo-controlled trial was conducted at outpatient clinics in Shanghai, China. Recruitment period: May 2021 through October 2024. Women aged 18–40 years with primary dysmenorrhea were randomized 1:1 to receive the Dysmenorrhea Patch (acupoint application patch) or a placebo patch for three consecutive menstrual cycles. Patches were applied to predefined acupoints for 8 hours daily during the non-menstrual phase of each cycle and withheld during menstruation. The primary endpoint was total effective rate (TER) based on the percentage reduction in Numerical Rating Scale (NRS) scores. Secondary endpoints included NRS, Cox Menstrual Symptom Scale (CMSS), Traditional Chinese Medicine (TCM) syndrome score, serum Prostaglandin F2α (PGF2α) and Prostaglandin E2 (PGE2), rescue ibuprofen use, and safety outcomes (local skin reactions and serum alanine aminotransferase [ALT]).

**Results:**

Of 110 randomized participants, 102 participants completed the end-of-treatment assessment. After three cycles, TER was significantly higher in the intervention group than the control group (60.00% vs. 34.55%, *p* = 0.013). Adjusted post-treatment NRS pain intensity was lower in the intervention group (3.08 ± 0.22 vs. 4.55 ± 0.22; *p* < 0.001). Menstrual pain duration showed a non-significant trend favoring the intervention (1.59 ± 0.07 vs. 1.76 ± 0.07 days; *p* = 0.087). The intervention group also demonstrated lower TCM syndrome scores (20.73 ± 1.85 vs. 27.98 ± 1.85; *p* = 0.007) and improved CMSS severity (12.82 ± 1.53 vs. 18.29 ± 1.53; *p* = 0.013) and duration scores (14.79 ± 1.51 vs. 20.41 ± 1.51; *p* = 0.010). Serum PGF2α decreased and PGE2 increased in the intervention group, with significant between-group differences for PGF2α (*p* = 0.027), PGE2 (*p* = 0.041), and PGF2α/PGE2 ratio (*p* < 0.001). Rescue ibuprofen use differed significantly between groups (*p* = 0.033), favoring the intervention. No adverse events or ALT abnormalities were observed.

**Conclusion:**

Dysmenorrhea Patch acupoint application for three menstrual cycles improved clinical response and reduced pain intensity and symptom burden in women with Qi stagnation and blood stasis type PD, with favorable prostaglandin changes and good safety. Larger and longer-term studies with rigorous assessment of blinding and concomitant medication effects are warranted.

**Clinical Trial Registration:**

https://www.chictr.org.cn/showprojEN.html?proj=60256, identifier ChiCTR2000037102.

## Background

Primary dysmenorrhea (PD) is the most prevalent gynecological complaint among menstruating women, with global prevalence rates ranging from 50% to 90% (1). PD is defined by cramp-like pain in the lower abdomen during menstruation, in the absence of identifiable pelvic pathology (2). PD significantly impairs daily functioning and quality of life, with approximately 20% of affected students missing classes and over 40% experiencing reduced concentration in the classroom (3).

Prostaglandins (PGs), particularly PGF2α and PGE2, play a central role in the pathogenesis of PD. PGF2α induces abnormal uterine smooth muscle contractions, leading to spasms, ischemia, hypoxia, and pain, while PGE2 relaxes uterine smooth muscle, inhibits its activity, and exerts analgesic effects (1, 4). Conventional treatments include non-steroidal anti-inflammatory drugs (NSAIDs), which alleviate symptoms by inhibiting prostaglandin synthesis (5). Despite their effectiveness, NSAIDs may cause adverse effects, such as gastrointestinal irritation, cardiovascular risks, and renal dysfunction, limiting their long-term use (6).

Consequently, many patients seek complementary and alternative therapies. Among these, external therapies in TCM—including acupuncture, moxibustion, tuina, and acupoint application—are widely used for dysmenorrhea treatment (7). Acupoint application involves the use of medicated plasters on specific acupoints to regulate qi and blood circulation, thereby alleviating pain (8). This method is simple, safe, and non-invasive, with minimal side effects, making it a viable option for patients seeking non-pharmacological pain relief (9, 10).

In Traditional Chinese Medicine, Qi stagnation and blood stasis is a commonly diagnosed syndrome associated with PD. To address this, Professor Zhu Nansun developed the classical Jiawei Mojie Decoction, which has been shown to be effective for treating Qi stagnation and blood stasis-type primary dysmenorrhea (11). The four key herbal ingredients—PuHuang (Typhae Pollen), MoYao (Myrrh), RuXiang (Frankincense), and XueJie (Dragon’s Blood)—are the main components of this decoction. Considering the convenience of acupoint application therapy, the patented Dysmenorrhea Patch was developed (12). This product incorporates the same key herbal ingredients for targeted application, providing a novel approach to treating dysmenorrhea. Preliminary studies have indicated that the Dysmenorrhea Patch effectively reduces blood viscosity and modulates the PGF2α/PGE2 balance in patients with dysmenorrhea. However, its efficacy and safety remain to be validated through large-scale clinical trials.

This study aimed to rigorously assess the efficacy and safety of the acupoint application with the Dysmenorrhea Patch in women with primary dysmenorrhea characterized by Qi stagnation and blood stasis. The findings may provide preliminary evidence supporting its use as an integrative treatment approach.

## Methods

### Study design and setting

This multicenter, randomized, double-blind, placebo-controlled superiority trial was conducted at three centers in Shanghai, China—Yueyang Hospital of Integrated Traditional Chinese and Western Medicine, Longhua Hospital affiliated with Shanghai University of Traditional Chinese Medicine, and the Shanghai Research Institute of Acupuncture and Meridian—from May 2021 through October 2024. Eligible participants were randomized in a 1:1 ratio to receive either the Dysmenorrhea Patch (intervention) or a matched placebo patch (control) for three consecutive menstrual cycles, with follow-up through the end of the third treatment cycle.

The intervention consisted of topical acupoint application with the Dysmenorrhea Patch, whereas the control group received an identically packaged placebo patch. All participants were permitted to use sustained-release ibuprofen (0.3 g per capsule) as rescue medication for intolerable pain, with all use recorded prospectively.

The primary outcome was the TER. Secondary outcomes included pain intensity (measured by NRS), pain duration, the CMSS, the TCM syndrome scale, serum PGF2α, PGE2, and the PGF2α/PGE2 ratio, as well as ibuprofen consumption. Serum ALT levels were measured for safety, and adverse events were recorded to assess the safety of the acupoint application therapy.

### Participants and sample size

Adult female participants aged 18–40 years were recruited if they met the following inclusion criteria: (1) Diagnosis of primary dysmenorrhea (ICD-10 N94.4) with a pain score ≥ 4 on the most painful day within the past 3 months; (2) Diagnosis of Qi stagnation and blood stasis syndrome according to TCM criteria (13); (3) Regular menstrual cycles (28 ± 7 days) lasting 3–7 days.

Exclusion criteria were as follows: (1) Secondary dysmenorrhea (e.g., endometriosis, adenomyosis); (2) severe, life-threatening comorbidities; (3) Psychiatric or neurological disorders (Self-Rating Anxiety Scale (SAS) or Self-Rating Depression Scale (SDS) score ≥ 50); (4) Current use of sedatives or hormonal medications; (5) Pregnancy, lactation, or allergies to the intervention patches.

Primary dysmenorrhea was diagnosed based on clinical history (cramping pelvic pain beginning near the onset of menses without evidence of pelvic pathology) and coded according to ICD-10 (N94.4). Secondary dysmenorrhea was excluded through a standardized gynecologic history and examination and pelvic ultrasonography performed within 1 month prior to enrollment (or at screening), as appropriate. All participants provided written informed consent before any study procedures were performed.

Participants were withdrawn from the study if they voluntarily withdrew their involvement, failed to complete the treatment protocol as scheduled, or used concomitant therapies for dysmenorrhea during the trial. Incomplete clinical documentation could also result in withdrawal. Treatment was discontinued if any serious adverse events (SAEs) or serious adverse reactions (SARs) occurred, or if participants developed severe intercurrent illnesses that prevented them from continuing the study. Pregnancy during the study period or failure to adhere to the study protocol were also grounds for termination.

The sample size was calculated for a superiority comparison of TER between groups. Based on our pilot data, the response rate was 77% in the intervention group and 45% in the control group. With a two-sided α of 0.05 and 80% power, 50 participants per group were required for a 1:1 allocation using a two-sample comparison of proportions. Allowing for an anticipated 10% attrition rate, the target sample size was set at 55 participants per group (110 total).

### Emotional disturbance assessment

Anxiety and depression can influence dysmenorrhea symptom severity. To minimize potential confounding from emotional factors, the SAS and SDS were used for eligibility screening. Participants with SAS ≥50 or SDS ≥50 were excluded. The SAS and SDS both consist of 20 items, with scores multiplied by 1.25 and rounded to determine the standard score. For the SAS, scores <50 indicate no anxiety, 50–59 indicate mild anxiety, 60–69 indicate moderate anxiety, and ≥70 indicate severe anxiety. For the SDS, scores <50 indicate no depression, 50–59 indicate mild depression, 60–69 indicate moderate depression, and ≥70 indicate severe depression.

### Randomization procedure and blinding

An independent statistician generated a 1:1 simple randomization sequence using Microsoft Excel 2010 with the RAND() function. The allocation list was password-protected and kept by the statistician. Study products were prepackaged by the manufacturer (Shanghai Wanshi Cheng Pharmaceutical Co., Ltd.) into identical kits and labeled only with a unique randomization number according to the allocation list. Investigators enrolled participants and assigned the next available randomization number in sequence. The corresponding kit was dispensed by pharmacy staff who were not involved in outcome assessment. The allocation code was not released until database lock. Participants, investigators, outcome assessors, and statistical analysts were blinded to group allocation. Emergency unblinding was permitted only when knowledge of the assigned treatment was essential for clinical management.

### Drug preparation

The Dysmenorrhea Patch was manufactured according to Chinese patent CN103520437A. The herbal components comprised Typhae Pollen (Puhuang), Frankincense (Ruxiang), Myrrh (Moyao), and Dragon’s Blood (Xuejie) in an approximately 15:5:5:5 ratio. The herbs were dispersed in a petrolatum-based matrix and coated onto a backing layer with standard excipients (e.g., dextrin, starch, and natural pigments). The placebo patch used the same backing layer and excipients but contained approximately 1/15 of the herbal content, with inert filler added to match appearance and texture and thereby maintain blinding. Both patches were produced by Shanghai Wanshi Cheng Pharmaceutical Co., Ltd.

### Study interventions

**Figure 1:**
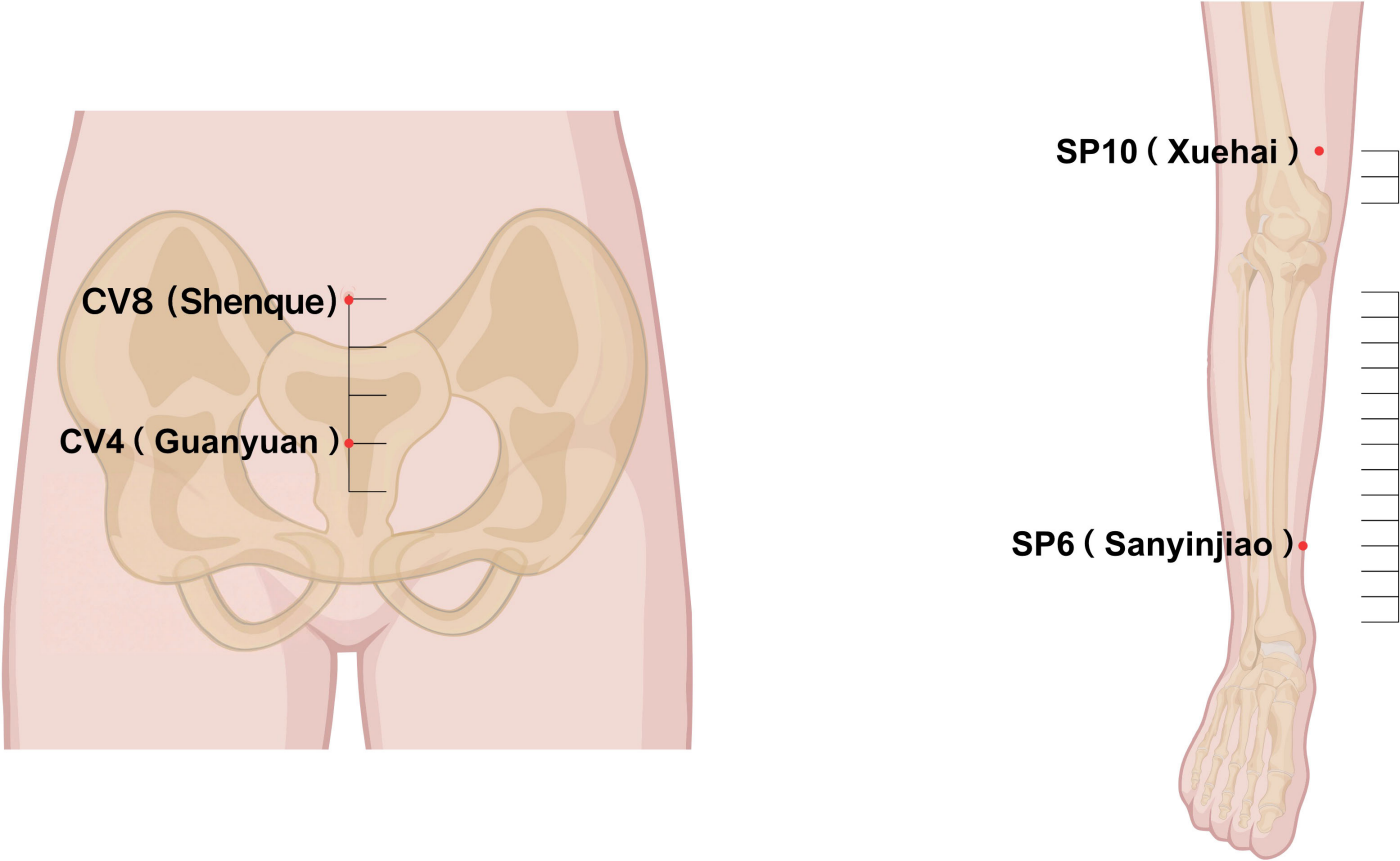
Location of the targeted acupoints.

All participants were provided with comprehensive instructions on the proper application of the intervention before the trial commenced. The intervention protocol was informed by traditional practice and the available clinical literature on acupoint application for primary dysmenorrhea (14–16). Four acupoints—SP6 (Sanyinjiao), SP10 (Xuehai), CV4 (Guanyuan), and CV8 (Shenque)—were chosen for their effectiveness in nourishing blood, regulating Qi, and relieving pain. The details of the location of the acupoints are shown in **Figure 1**. The “Dysmenorrhea Patch” was applied to these acupoints following skin disinfection with 75% ethanol. A patch was placed on each acupoint, worn for eight hours daily, preferably overnight, and removed in the morning. Patches were applied daily from the day after menstruation ended until the day before the next menstruation; no patches were applied during menstruation. The control group received identical placebo patches, applied in the same manner. Adherence was promoted through standardized instructions at enrollment and reminders during follow-up. Participants recorded daily application in a diary, and study staff verified adherence at each visit by reviewing the diary and patch usage records.

Local skin reactions (e.g., erythema, pruritus, rash, or blistering) were monitored throughout the study. Participants were instructed to discontinue patch application temporarily if moderate irritation occurred and to contact study staff for assessment; permanent discontinuation was recommended if symptoms were severe or persistent.

### Measurements

#### Primary outcome

The primary outcome was TER, which was calculated from the reduction rate in NRS pain scores; clinical efficacy was classified into four levels (17):

Clinical cure (reduction rate ≥ 95%),

Markedly effective (70% ≤ reduction rate < 95%),

Generally effective (30% ≤ reduction rate < 70%),

Ineffective (reduction rate < 30%).

Total effective rate (%) = (Number of cases with clinical cure + Number of cases with markedly effective response + Number of cases with generally effective response)/Total number of cases × 100%.

#### Secondary outcomes

##### The Numerical Rating Scale

NRS is a validated, unidimensional tool for assessing pain intensity, where patients rate their pain from 0 (“no pain”) to 10 (“worst imaginable pain”) (18). NRS provides a simple and effective measure of menstrual pain severity and its impact on daily activities (19).

##### Duration of dysmenorrhea

Due to variability in the duration of dysmenorrhea among participants, the number of days with menstrual pain was recorded. Changes in pain duration were analyzed to evaluate treatment efficacy.

##### Cox Menstrual Symptom Scale

CMSS, a validated 18-item questionnaire scored on a five-point Likert scale, was used to assess the severity and duration of menstrual pain and related symptoms (20).

##### Traditional Chinese Medicine Syndrome Score

A standardized TCM syndrome scale was applied to evaluate dysmenorrhea-related symptoms according to the Guidelines for Diagnosis and Treatment of Common Gynecological Diseases in TCM (2012) (21). The scale comprises 24 items scored from 0 to 3 (absent to severe), covering menstrual pain characteristics, menstrual features, and systemic symptoms, with a total score range of 0–72. Higher scores indicate more pronounced TCM-related manifestations. Reliability testing showed Cronbach’s α > 0.8, indicating good internal consistency.

##### Serum prostaglandin levels (PGF2α and PGE2) and the PGF2α/PGE2 ratio

PGF2α and PGE2 were measured, as excessive PGF2α induces uterine hypercontractility and ischemic pain, while PGE2 can relax uterine smooth muscle. The PGF2α/PGE2 ratio was used as an objective biomarker to assess dysmenorrhea severity and treatment efficacy (22). To evaluate potential confounding by rescue analgesics, prostaglandin outcomes (PGF2α, PGE2, and PGF2α/PGE2 ratio) were re-analyzed after excluding participants who used rescue ibuprofen during the study period.

##### Ibuprofen consumption

Ibuprofen sustained-release capsules (0.3 g) were allowed as rescue medication when menstrual pain became intolerable. A maximum of three doses within 24 hours was permitted, with at least 8 hours between doses. Rescue ibuprofen consumption was defined as the total number of doses taken during the dysmenorrhea period of each menstrual cycle and was recorded in the case report form.

### Safety assessment

Adverse events, including potential skin reactions such as erythema, rash, or pruritus, were documented, with details of onset, duration, management, and resolution. Serum ALT was assessed at baseline and post-treatment to monitor hepatic safety. Although topical Chinese herbal therapy is primarily intended for local effects, conservative liver enzyme monitoring was performed in view of potential transdermal absorption and the risk of herb-related liver injury.

### Data collection

Outcome assessments, including dysmenorrhea peak pain intensity, pain duration, rescue ibuprofen consumption, CMSS, and TCM syndrome scores, were performed at two time points: baseline (enrollment) and post-treatment (after three menstrual cycles). Ibuprofen consumption was recorded throughout each cycle. Peripheral blood samples were collected twice during days 2–7 of menstruation: once at baseline (pre-treatment) and once at the end-of-treatment (after three treated cycles).

### Data analysis

All analyses followed the intention-to-treat principle and were performed using SPSS 26.0 and the PROCESS macro (Version 4.3). Continuous variables were summarized as mean ± SD or median (IQR) as appropriate, and categorical variables as n (%). A two-sided *p* < 0.05 was considered statistically significant.

The primary endpoint (TER) was tested at a two-sided α = 0.05. For prespecified key secondary outcomes (NRS pain intensity, pain duration, CMSS severity, CMSS duration, and TCM syndrome score), Holm–Bonferroni adjusted *p* values were additionally reported (**Supplementary Table S1**) to control the family-wise error rate (m = 5). Other secondary outcomes were interpreted as exploratory.

Missing post-treatment questionnaire outcomes were handled using multiple imputation. ANCOVA models were used to compare post-treatment outcomes between groups while adjusting for the corresponding baseline value. Homogeneity of regression slopes was assessed using the group×baseline interaction; when non-significant, the interaction term was removed and adjusted means and 95% confidence intervals were reported. Bootstrap validation (1000 resamples; BCa 95% CI) was applied for key ANCOVA estimates.

Moderation analyses for CMSS severity and duration were conducted using PROCESS Model 1 with mean-centered baseline scores as moderators; conditional effects and Johnson–Neyman regions were used to identify baseline thresholds associated with statistically significant treatment effects.

Prostaglandin biomarkers (PGF2α, PGE2, and PGF2α/PGE2 ratio) and rescue ibuprofen use were analyzed using non-parametric methods: within-group changes were assessed by Wilcoxon signed-rank tests and between-group comparisons by Mann–Whitney U tests. A prespecified sensitivity analysis excluding participants who used rescue ibuprofen during treatment was performed for prostaglandin outcomes.

### Ethical considerations

The hospital ethics committee approved the clinical study protocol, informed consent form, subject recruitment advertisement, case report form (CRF), subject identification code form, principal investigator’s curriculum vitae, and training certificate (approval number: KYSKSB2020-136). The registration number for this study was ChiCTR2000037102.

## Results

### Participant characteristics

**Figure 2:**
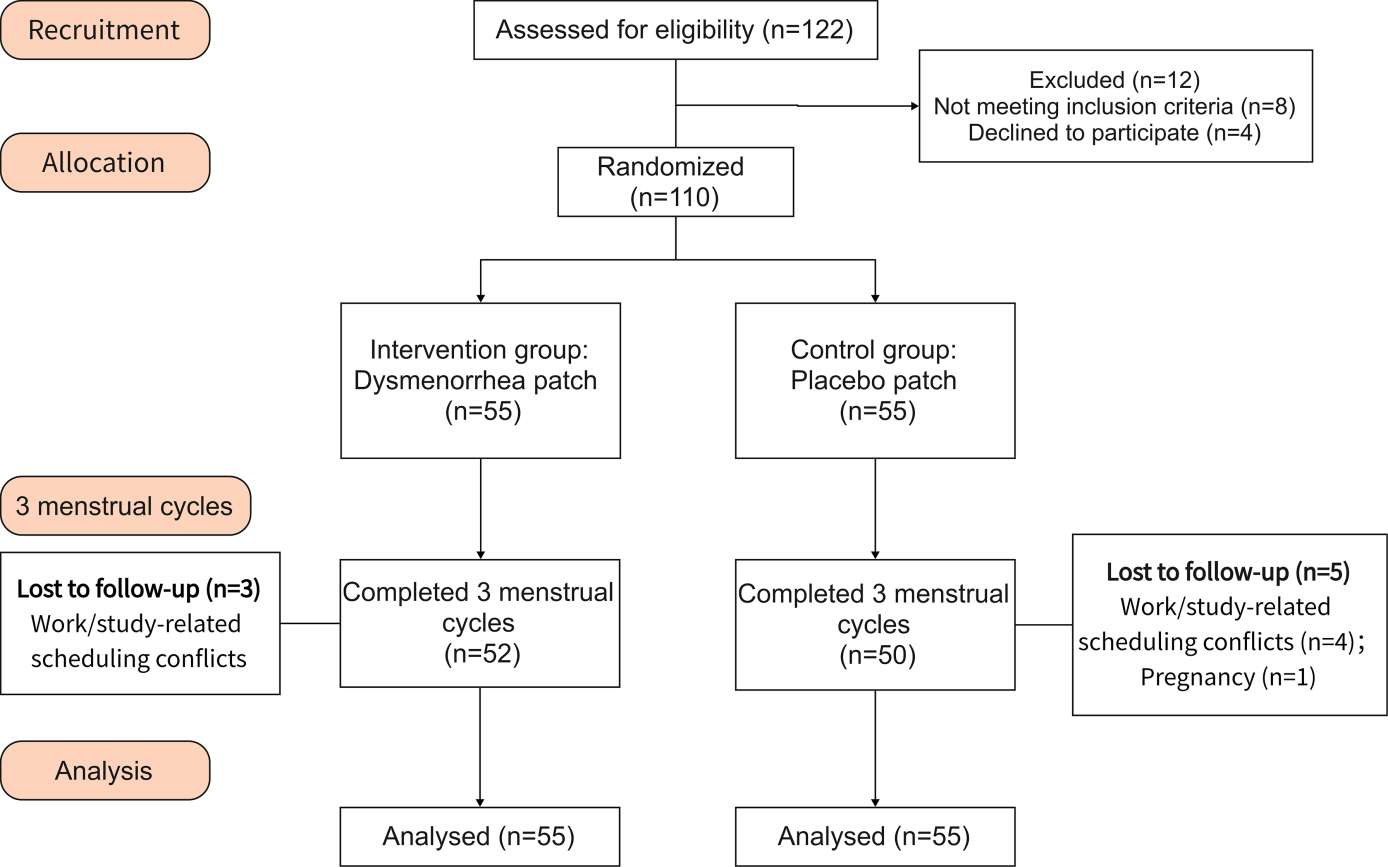
CONSORT Flowchart.

Of the 122 patients screened, 12 were excluded because they did not meet the inclusion criteria or declined to participate. A total of 110 participants were randomized. In the intervention group, 3 participants did not complete the end-of-treatment assessment because work- or study-related scheduling conflicts prevented attendance for follow-up visits and blood sampling. In the control group, 4 participants discontinued for the same reason, and 1 participant withdrew due to pregnancy. Therefore, 52 participants in the intervention group and 50 participants in the control group completed the end-of-treatment visit and provided paired blood samples for prostaglandin analyses. At study completion, among participants who completed the blinding questionnaire (intervention: n=52; control: n=50), 53.85% of participants in the intervention group and 50.00% in the control group reported “don’t know”; correct guesses were 26.92% and 24.00%, suggesting blinding was generally maintained. **Figure 2** presents the trial CONSORT flow diagram. The demographic and clinical characteristics at baseline were comparable between the groups, indicating the adequacy of the randomization process (**Table 1**).

**Table 1:** Characteristics of participants at baseline.

Variables	Intervention group(n=55)	Control group(n=55)	*Z*/*t*/χ*^2^*	*p*
Age, median (IQR)	25.00(6.00)	25.00(6.00)	-0.048	0.962 ^a^
BMI, mean (SD)	20.25(2.62)	20.61(2.43)	-0.745	0.458 ^b^
Age at Menarche, median (IQR)	13.00(2.00)	13.00(2.00)	-0.221	0.825 ^a^
Cycle Length, median (IQR)	29.00(3.00)	30.00(2.00)	-0.027	0.978 ^a^
Days of Menstruation, median (IQR)	6.00(2.00)	6.00(2.00)	-1.082	0.279 ^a^
Dysmenorrhea Duration, median (IQR)	8.00(5.00)	9.00(8.00)	-0.619	0.536 ^a^
Pain Intensity, median (IQR)	5.00(2.00)	5.00(3.00)	-0.38*8*	0.698 ^a^
Pain Duration, median (IQR)	2.00(1.00)	2.00(1.00)	-0.293	0.769 ^a^
CMSS Severity Score, median (IQR)	18.00(14.00)	16.00(19.00)	-0.281	0.779 ^a^
CMSS Duration Score, median (IQR)	20.00(15.00)	20.00(15.00)	-0.404	0.686 ^a^
CMSS Total Score, median (IQR)	39.00(26.00)	36.00(32.00)	-0.538	0.590 ^a^
TCM Syndrome Score, mean (SD)	28.64(11.29)	26.87(13.13)	0.755	0.452 ^b^
SAS Score, mean (SD)	33.95(6.74)	33.91(6.77)	-0.028	0.978 ^b^
SDS Score, mean (SD)	30.85(4.86)	30.76(4.96)	-0.097	0.923 ^b^
Serum PGF2α, median (IQR)	21.16(10.52)	18.57(8.08)	-1.292	0.196 ^a^
Serum PGE2, median (IQR)	290.52(167.56)	259.44(146.34)	-0.596	0.551 ^a^
PGF2α/PGE2 Ratio, median (IQR)	0.08(0.04)	0.07(0.04)	-0.736	0.462 ^a^
Educational level [n(%)]				
College or Equivalent	36(65.45)	28(50.91)	2.391	0.122 ^c^
Graduate or Higher	19(34.55)	27(49.09)		
Sexual history [n(%)]				
No	29(52.73)	25(45.45)	0.582	0.446 ^c^
With	26(47.27)	30(54.55)		
Obstetric History [n(%)]				
No	52(94.55)	51(92.73)	0.153	0.696 ^c^
With	3(5.45)	4(7.27)		

BMI Body mass index, CMSS Cox Menstrual Symptom Scale, TCM Traditional Chinese Medicine, SAS Self-Rating Anxiety Scale, SDS Self-Rating Depression Scale.

^a^Mann–Whitney U test.

^b^Independent-samples t test.

^c^Chi-squared test.

### Primary outcomes

The primary analysis followed the intention-to-treat principle and included all randomized participants (n=110). TER was derived from the percentage reduction in NRS from baseline to the end of the third treated cycle according to the prespecified criteria. For participants without an end-of-treatment NRS assessment, the end-of-treatment NRS was imputed using multiple imputation incorporating baseline NRS, the last observed NRS prior to discontinuation, and the timing of the last assessment (cycle number). After three menstrual cycles, the intervention group exhibited a significantly higher TER than the control group (60.00% vs. 34.55%, *p* = 0.013, **Table 2**). Sensitivity analyses across different analysis populations yielded consistent results. In the intention-to-treat (ITT) analysis using last observation carried forward (LOCF), TER remained significantly higher in the intervention group than in the control group (60.00% vs. 30.91%; *p* = 0.002). Similarly, in the per-protocol (PP) population excluding dropouts, the intervention group maintained a significantly higher TER compared with the control group (59.62% vs. 34.00%; *p* = 0.010), supporting the robustness of the primary efficacy finding. (**Supplementary Table S2**)

**Table 2:** Comparison of treatment efficacy between the two groups.

Group	Total effective				Ineffective		χ^2^	*p*
	Clinical cure (n)	Markedly effective (n)	Generally effective (n)	%	Cases	%		
Intervention Group(n=55)	2	10	21	60.00	22	40.00	7.149	0.013
Control Group(n=55)	0	4	15	34.55	36	65.45		

### Secondary outcomes

Secondary outcomes were analyzed in the intention-to-treat population (n=55 per group). After adjusting for baseline values, the intervention group showed lower post-treatment NRS pain intensity than the control group (adjusted mean ± SE: 3.08 ± 0.22 vs. 4.55 ± 0.22), corresponding to an adjusted mean difference (I-C) of -1.47 (*p* < 0.001). Bootstrap validation (1000 resamples) supported the robustness of this finding (BCa 95% CI -2.08 to -0.84; *p* < 0.001).

For menstrual pain duration (days), the intervention group showed a non-significant trend toward fewer pain days than the control group (adjusted mean ± SE: 1.59 ± 0.07 vs. 1.76 ± 0.07), with an adjusted difference (I-C) of -0.17 (*p* = 0.087). Bootstrap analysis yielded consistent results (BCa 95% CI -0.37 to 0.01; *p* = 0.098).

Post-treatment TCM syndrome scores were also lower in the intervention group after adjusting for baseline (adjusted mean ± SE: 20.73 ± 1.85 vs. 27.98 ± 1.85), corresponding to an adjusted difference (I-C) of -7.26 (*p* = 0.007). Bootstrap validation confirmed the between-group difference (BCa 95% CI -12.47 to -2.24; *p* = 0.008) (**Table 3**, **Supplementary Table S3**).

**Table 3:** Effects of the NRS, menstrual pain duration and TCM syndrome scale at post-treatment.

Outcome	Adjusted mean ± SE		Adjusted mean difference (I-C)	95% CI	*p*	Partial η²
	Intervention (n=55)	Control (n=55)				
NRS pain intensity	3.08 ± 0.22	4.55 ± 0.22	-1.47	-2.08 to -0.86	<0.001	0.176
Pain duration (days)	1.59 ± 0.07	1.76 ± 0.07	-0.17	-0.36 to 0.03	0.087	0.027
TCM syndrome score	20.73 ± 1.85	27.98 ± 1.85	-7.26	-12.45 to -2.07	0.007	0.067

Values are estimated marginal means (EMMs) ± SE from ANCOVA models adjusting for baseline (baseline mean-centered). (I−C) indicates the adjusted post-treatment difference; negative values favor the intervention. Partial η² indicates effect size.

For CMSS outcomes, adjusted post-treatment scores were lower in the intervention group than in the control group for both severity (12.82 ± 1.53 vs. 18.29 ± 1.53; adjusted difference [I−C] = −5.47, 95% CI −9.77 to −1.17; *p* = 0.013) and duration (14.79 ± 1.51 vs. 20.41 ± 1.51; adjusted difference [I−C] = −5.63, 95% CI −9.87 to −1.38; *p* = 0.010). Moderation analyses (PROCESS Model 1) indicated that baseline CMSS severity and duration significantly moderated treatment effects (interaction *p* = 0.049 and *p* = 0.005, respectively) (**Table 4**). Johnson–Neyman analyses identified raw baseline thresholds of 16.12 (severity) and 17.80 (duration), above which the intervention demonstrated significantly greater symptom improvement than control (**Supplementary Tables S4, S5**).

**Table 4:** Effects of the CMSS score at post-treatment.

Outcome (post-treatment)	Adjusted mean ± SE		Adjusted mean difference (I-C)	95% CI	*p*	Partial η²	Interaction p (Group×Baseline)
	Intervention (n=55)	Control (n=55)					
CMSS severity score	12.82 ± 1.53	18.29 ± 1.53	-5.47	-9.77 to -1.17	0.013	0.057	0.049
CMSS duration score	14.79 ± 1.51	20.41 ± 1.51	-5.63	-9.87 to -1.38	0.010	0.061	0.005

Values are estimated marginal means (EMMs) ± SE from ANCOVA models adjusting for baseline (baseline mean-centered). Negative (I−C) indicates lower post-treatment scores in the intervention group. Interaction *p* values refer to the group×baseline term.

Changes in prostaglandin biomarkers were observed during treatment. In the intervention group, serum PGF2α decreased from 21.16 (10.52) to 19.59 (10.45) (*Z* = -2.468, *p* = 0.014), whereas no significant change was detected in the control group (18.57 (8.08) to 20.39 (11.33), *Z* = -1.742, *p* = 0.081); the between-group comparison indicated a significant difference in PGF2α change favoring the intervention (*Z* = -2.209, *p* = 0.027). Serum PGE2 increased in the intervention group (290.52 (167.56) to 388.89 (469.00), *Z* = -3.351, *p* < 0.001) but not in the control group (259.44 (146.34) to 289.60 (175.89), *Z* = -1.028, *p* = 0.304), with a significantly greater increase in the intervention group (*Z* = -2.042, *p* = 0.041). Accordingly, the PGF2α/PGE2 ratio decreased in the intervention group (0.08 (0.04) to 0.06 (0.06), *Z* = -3.488, *p* < 0.001), while remaining unchanged in the control group (0.07 (0.04) to 0.07 (0.04), *Z* = -0.671, *p* = 0.502); the reduction was significantly larger in the intervention group (*Z* = -3.528, *p* < 0.001) (**Table 5**). A prespecified sensitivity analysis excluding participants who used rescue ibuprofen showed consistent findings, with significant post-treatment between-group differences in PGE2 (*p* = 0.019) and PGF2α/PGE2 ratio (*p* = 0.002), while PGF2α showed a non-significant trend (*p* = 0.083) (**Supplementary Table S6**).

**Table 5:** Changes in prostaglandin biomarkers and rescue ibuprofen use.

Outcome	Group	Before Treatment	After Treatment	*Z*	*p*
Serum PGF2α, median (IQR)	Intervention Group(n=52)	21.16(10.52)	19.59(10.45)	-2.468	0.014^a^
	Control Group(n=50)	18.57(8.08)	20.39(11.33)	-1.742	0.081^a^
	Between-group difference			-2.209	0.027^b^
Serum PGE2, median (IQR)	Intervention Group(n=52)	290.52(167.56)	388.89(469.00)	-3.351	<0.001^a^
	Control Group(n=50)	259.44(146.34)	289.60(175.89)	-1.028	0.304^a^
	Between-group difference			-2.042	0.041^b^
PGF2α/PGE2 Ratio, median (IQR)	Intervention Group(n=52)	0.08(0.04)	0.06(0.06)	-3.488	<0.001^a^
	Control Group(n=50)	0.07(0.04)	0.07(0.04)	-0.671	0.502^a^
	Between-group difference			-3.528	<0.001^b^
Ibuprofen usage, median (IQR)	Intervention Group(n=27)	1.00(1.00)	1.00(2.00)	-4.123	<0.001^a^
	Control Group(n=29)	1.00(1.00)	1.00(1.00)	-1.732	0.083^a^
	Between-group difference			-2.133	0.033^b^

^a^Wilcoxon signed-rank test.

^b^Mann–Whitney U test. n varies due to availability of paired samples/ibuprofen users only.

Exploratory analyses of sex hormones measured on cycle days 2–7 are summarized in **Supplementary Table S7**. Nominal Group×Time interactions were observed for progesterone and testosterone (and a trend for FSH), but none remained statistically significant after Benjamini-Hochberg False Discovery Rate (BH-FDR) correction across six hormones (all *q*≥0.134).

Rescue ibuprofen use was summarized as the total number of doses taken during the dysmenorrhea period of each menstrual cycle. Among participants who used ibuprofen at least once (intervention: 27/55, 49.1%; control: 29/55, 52.7%), the intervention group showed a significant reduction in ibuprofen use from baseline to post-treatment (1.00 (1.00) to 1.00 (2.00); *Z* = -4.123, *p* < 0.001), whereas the control group showed no significant change (1.00 (1.00) to 1.00 (1.00); *Z* = -1.732, *p* = 0.083). Post-treatment ibuprofen use differed significantly between groups (*Z* = -2.133, *p* = 0.033), indicating lower analgesic demand in the intervention group (**Table 5**). To further describe usage patterns, we additionally summarized (i) the proportion of participants using any ibuprofen per cycle and (ii) the distribution of dose counts (0, 1, 2, ≥3 doses) at baseline and post-treatment (**Supplementary Table S8**).

### Safety assessment

No adverse events were reported during the treatment period. Serum levels of ALT were measured in both the intervention and control groups before and after treatment. No abnormalities were observed in either group.

## Discussion

This study aimed to evaluate the efficacy and safety of acupoint application therapy for Qi stagnation and blood stasis type primary dysmenorrhea. The primary endpoint, TER, was significantly higher in the intervention group than in the control group after three menstrual cycles of treatment. This supports the overall clinical effectiveness of the Dysmenorrhea Patch for Qi stagnation and blood stasis type PD with access to rescue medication as needed.

Regarding secondary outcomes, ANCOVA-adjusted analyses further indicated that NRS was significantly lower in the intervention group than in the control group at post-treatment, reflecting clinically meaningful analgesic benefit.

For menstrual pain duration, the intervention group showed a trend toward fewer pain days compared with the control group; however, this difference did not reach statistical significance in adjusted analyses. This pattern suggests that the patch may more strongly affect peak pain intensity and overall symptom burden than the number of pain days within the current follow-up window. In addition, the study was powered for a larger effect size than ultimately observed, which may partly explain why smaller between-group differences—such as pain duration—did not reach significance. Future trials with larger sample sizes or longer follow-up may better clarify effects on duration-related outcomes.

Beyond pain intensity, the intervention improved broader symptom burden. Post-treatment TCM syndrome scores were significantly lower in the intervention group after baseline adjustment, indicating improvement in TCM-relevant symptom patterns and overall dysmenorrhea manifestations. CMSS severity and duration scores were also significantly improved in the intervention group, suggesting benefits on both the intensity and time-course of dysmenorrhea-related symptoms captured by a validated multidimensional scale.

A notable finding is that baseline symptom severity moderated treatment effects on CMSS outcomes. Moderation analyses identified baseline thresholds above which the intervention demonstrated significantly greater symptom improvement than the control. Clinically, this implies that patients with higher baseline symptom burden may derive greater benefit from acupoint application with the Dysmenorrhea Patch. One interpretation is that when symptoms are mild, contextual factors and natural variability may account for a larger proportion of perceived improvement, whereas in more severe cases the pharmacological and physiological contributions of the intervention may become more apparent. This “greater benefit in more severe PD” pattern warrants replication and may inform stratified clinical recommendations.

Improvements observed within the control group should not be interpreted as purely a placebo effect, nor should within-group changes be used to infer treatment efficacy. In PD trials, symptom scores can fluctuate naturally across cycles, and changes can reflect contextual factors (e.g., clinical contact, expectancy), self-management behaviors during follow-up, and regression to the mean (23–25). In addition, to maintain blinding fidelity, the placebo patch contained a small fraction of the active herbal content (approximately 6.7%), which may have introduced a mild pharmacological effect (26). Therefore, the most appropriate inference should emphasize adjusted between-group comparisons and effect estimates rather than attributing within-group changes to “therapeutic” versus “placebo” mechanisms.

Because this study enrolled women with primary dysmenorrhea and excluded secondary causes through clinical evaluation and pelvic ultrasonography at screening, the findings should be interpreted as evidence for PD rather than generalized to secondary dysmenorrhea (e.g., endometriosis or adenomyosis). Secondary dysmenorrhea often involves structural and inflammatory etiologies that may not respond similarly to an acupoint application approach. Dedicated studies would be required to evaluate efficacy in secondary dysmenorrhea populations.

From a biomedical perspective, PD is closely related to prostaglandin dysregulation, uterine hypercontractility, and ischemia/hypoxia-related nociception. Excessive PGF2α contributes to strong uterine contractions and vasoconstriction, while PGE2 can exert more complex effects including smooth muscle relaxation in certain contexts; thus, the balance between these mediators may influence uterine activity and pain perception (27). In this trial, the intervention was associated with decreased PGF2α, increased PGE2, and a reduced PGF2α/PGE2 ratio, a pattern consistent with a less spastic and less ischemic uterine environment.

Sex hormones were assessed on cycle days 2–7 at baseline and after three treated cycles. Although progesterone and testosterone showed nominal Group×Time interactions, none of these findings remained statistically significant after BH-FDR correction, indicating that the observed differences may reflect variability inherent to endocrine measurements, limited power for mechanistic endpoints, and multiplicity across hormones. Importantly, LH, estradiol, and prolactin remained broadly stable between groups over time, which argues against a large shift in the hypothalamic–pituitary–ovarian axis within the studied window. In this context, the hormonal findings are best interpreted as exploratory and hypothesis-generating; they do not materially alter the primary interpretation that the intervention was associated with clinical benefit, while the prostaglandin-related results provide a more proximal mechanistic signal to be examined in future work.

From a TCM perspective, Qi stagnation and blood stasis is a common pattern in dysmenorrhea, often corresponding to distending or stabbing pain that intensifies around menstruation. Acupoint application is intended to regulate Qi, activate blood circulation, and relieve pain. The patch’s core herbal components have traditionally been used for “activating blood and relieving pain,” and modern pharmacological evidence supports their anti-inflammatory and analgesic potential (9, 27, 28). Together, the symptom improvements and biomarker shifts observed in this study provide an integrative bridge between TCM pattern-based rationale and plausible biomedical pathways.

Rescue ibuprofen consumption is clinically relevant because it reflects analgesic demand under real-world conditions. In the present trial, approximately half of participants used rescue ibuprofen at least once, and consumption was summarized as the total number of doses taken during the dysmenorrhea period of each menstrual cycle. The intervention group exhibited a greater reduction in rescue ibuprofen use than the control group, with a significant between-group difference at post-treatment. Together with improvements in pain intensity and symptom burden, the reduced reliance on rescue ibuprofen suggests that the acupoint application may offer an analgesic-sparing effect. Because rescue ibuprofen use differed between groups and can directly influence cyclooxygenase pathways, prostaglandin biomarkers should be interpreted cautiously, as they may be partly affected by NSAID exposure (29). Accordingly, we interpreted the biomarker findings alongside analgesic-use patterns and the prespecified sensitivity analyses that excluded participants who used rescue ibuprofen. The similar direction of results in these analyses suggests that NSAID exposure alone is unlikely to account for the between-group biomarker differences. Nevertheless, variations in timing and dose of rescue medication across individuals may still affect prostaglandin dynamics; future studies should consider standardizing the time window between the last rescue dose and blood sampling and adjusting for cycle-level ibuprofen exposure to strengthen causal inference.

Psychological and lifestyle factors (e.g., stress, sleep disturbance, anxiety, and depressive symptoms) can influence pain perception and symptom reporting in PD. In the present study, SAS and SDS were used primarily for screening of clinically significant anxiety or depression rather than as longitudinal outcomes. Accordingly, the trial was not designed to test whether changes in psychological factors mediated treatment response. Future studies should incorporate repeated assessments of psychological variables across cycles and explore mediation or moderation effects to clarify interactions between emotional factors and symptom improvement.

No adverse events were reported during the treatment period, and no abnormalities were observed in serum ALT before versus after treatment in either group, supporting overall tolerability of the intervention. Although systemic absorption from topical application is expected to be limited, ALT was included as a conservative safety biomarker because the intervention contains multiple herbal constituents and was applied repeatedly across three cycles. Monitoring liver enzymes represents a prudent pharmacovigilance measure in herbal-product trials to detect unexpected systemic exposure or idiosyncratic reactions, in addition to documenting local skin reactions such as erythema, rash, or pruritus (30).

### Strengths

This trial adopted a multicenter, randomized, double-blind, placebo-controlled design, improving internal validity. Key outcomes combined patient-centered measures (pain intensity, symptom burden, and rescue medication use) with mechanistic biomarkers (PGF2α, PGE2, and PGF2α/PGE2 ratio), allowing a more integrated interpretation of clinical and biological effects. The study also applied an intention-to-treat framework with multiple imputation and prespecified sensitivity analyses to support robustness.

### Limitations

The follow-up period was limited to three menstrual cycles, and longer-term follow-up is warranted to evaluate the durability of benefit and recurrence patterns beyond this period. Lifestyle and psychological factors were not measured longitudinally, limiting our ability to evaluate their potential contributions to pain outcomes. Future studies could incorporate repeated assessments of these covariates throughout follow-up. Although we restricted enrollment to women aged 18–40 years, residual age-related heterogeneity may still influence symptom trajectories and treatment response, and larger trials could consider stratified randomization and/or prespecified age subgroup analyses. To preserve masking, the placebo patch used identical excipients and a minimal (1/15) herbal content to enhance sensory similarity. This design may have reduced the apparent between-group contrast and may limit attribution solely to the full-dose patch; future studies could consider optimizing placebo design. Finally, while sensitivity analyses addressed potential confounding from rescue ibuprofen in prostaglandin biomarkers, future studies could further strengthen mechanistic inference by standardizing sampling relative to NSAID exposure and/or applying additional modeling approaches.

## Conclusion

In women with Qi stagnation and blood stasis type primary dysmenorrhea, the Dysmenorrhea Patch significantly reduces pain intensity, improves associated symptoms and TCM syndrome manifestations, and reduces the use of analgesics; a non-significant trend toward reduced pain duration was observed. The Dysmenorrhea Patch is well tolerated, supporting its efficacy and safety in this population. The Dysmenorrhea Patch may exert its therapeutic effects by lowering serum PGF2α levels, increasing serum PGE2 levels, and regulating the PGF2α/PGE2 ratio, thus treating Qi stagnation and blood stasis type primary dysmenorrhea.

## Data Availability

The datasets presented in this article are not readily available because The original data has been archived by the hospital. Requests to access the datasets should be directed to Chun Yan Zhang, zh_ch_yan@163.com.
