# Supplementary material for: Efficacy and safety of Dysmenorrhea Patch acupoint application in women with primary dysmenorrhea: a randomized double-blind controlled trial
Source: Front Endocrinol (Lausanne). 2026 Feb 25;17:1728199. doi: 10.3389/fendo.2026.1728199 (PMC12975456; doi:10.3389/fendo.2026.1728199)
Supplement: Supplementary file 1 [file Table1.docx]

Supplementary Table S1. Holm–Bonferroni adjusted p values for prespecified key secondary outcomes

| Prespecified key secondary outcome (post-treatment, baseline-adjusted) | Raw *p* | Holm–Bonferroni adjusted *p* |
| --- | --- | --- |
| NRS pain intensity | 0.000006 | 0.000030 |
| TCM syndrome score | 0.007 | 0.028 |
| CMSS duration | 0.010 | 0.030 |
| CMSS severity | 0.013 | 0.030 |
| Pain duration | 0.087 | 0.087 |

Holm–Bonferroni procedure was applied across prespecified key secondary outcomes (m = 5) to control the family-wise error rate at α = 0.05. Raw p values correspond to the between-group comparison at post-treatment with baseline adjustment (ANCOVA). Adjusted p values were computed using the step-down Holm method and are reported after enforcing monotonicity.

Supplementary Table S2. Sensitivity Analysis of Total Effective Rate across Different Populations

| Analysis Population | Group | N | Total Effective [n (%)] | Clinical Cure (n) | Markedly Effective (n) | Generally Effective (n) | Ineffective [n (%)] | χ2 | *p* |
| --- | --- | --- | --- | --- | --- | --- | --- | --- | --- |
| ITT (LOCF) | Intervention | 55 | 33 (60.00%) | 2 | 10 | 21 | 22 (40.00%) | 9.387 | 0.002 |
|  | Control | 55 | 17 (30.91%) | 0 | 3 | 14 | 38 (69.09%) |  |  |
| PP (Dropouts Excluded) | Intervention | 52 | 31 (59.62%) | 2 | 10 | 19 | 21 (40.38%) | 6.713 | 0.010 |
|  | Control | 50 | 17 (34.00%) | 0 | 3 | 14 | 33 (66.00%) |  |  |

Note: ITT = Intention-to-Treat; LOCF = Last Observation Carried Forward; PP = Per-Protocol. Differences between groups were analyzed using the Chi-square test.

Supplementary Table S3. ANCOVA-adjusted effects on secondary outcomes with bootstrap validation (n=55 per group)

| Outcome (post-treatment) | Interaction (Group×Baseline) *p* | Adjusted mean ± SE (Intervention) | Adjusted mean ± SE (Control) | Adjusted mean difference (I−C) | Conventional 95% CI (difference) | *p* (ANCOVA) | Partial η² | Bootstrap *p* | BCa 95% CI (difference) |
| --- | --- | --- | --- | --- | --- | --- | --- | --- | --- |
| NRS pain intensity | 0.372 | 3.08 ± 0.22 | 4.55 ± 0.22 | −1.47 | −2.08 to −0.86 | <0.001 | 0.176 | <0.001 | −2.08 to −0.84 |
| Menstrual pain duration (days) | 0.348 | 1.59 ± 0.07 | 1.76 ± 0.07 | −0.17 | −0.36 to 0.03 | 0.087 | 0.027 | 0.098 | −0.37 to 0.01 |
| TCM syndrome score | 0.113 | 20.73 ± 1.85 | 27.98 ± 1.85 | −7.26 | −12.45 to −2.07 | 0.007 | 0.067 | 0.008 | −12.47 to −2.24 |

Values are estimated marginal means (EMMs) ± standard error (SE) from ANCOVA models adjusting for the corresponding baseline value.

The homogeneity of regression slopes assumption was assessed using the group×baseline interaction; as interaction terms were non-significant (*p* > 0.05), they were removed from the final models. Bootstrap validation was performed using 1000 resamples with bias-corrected and accelerated (BCa) 95% confidence intervals. Negative adjusted mean differences (I−C) indicate lower post-treatment scores in the intervention group (favoring the intervention).

Abbreviations: ANCOVA, analysis of covariance; BCa, bias-corrected and accelerated; CI, confidence interval; EMM, estimated marginal mean; NRS, Numerical Rating Scale; SE, standard error; TCM, traditional Chinese medicine.

Supplementary Table S4. Bootstrap validation for CMSS outcomes

| Outcome (post-treatment) | Adjusted mean difference (I−C) | Conventional 95% CI | *p* (ANCOVA) | Bootstrap *p* | BCa 95% CI (bootstrap) |
| --- | --- | --- | --- | --- | --- |
| CMSS severity score | −5.47 | −9.77 to −1.17 | 0.013 | 0.022 | −10.51 to −0.61 |
| CMSS duration score | −5.63 | −9.87 to −1.38 | 0.010 | 0.012 | −9.95 to −1.29 |

Bootstrap used bias-corrected and accelerated (BCa) 95% confidence intervals with 1000 resamples; results were consistent with the primary ANCOVA.

Supplementary Table S5. Baseline-moderated treatment effects on CMSS outcomes (PROCESS Model 1; mean-centered baseline)

1. CMSS severity

| Moderator level | Baseline severity (raw) | Effect (Control − Intervention) | 95% CI | *p* |
| --- | --- | --- | --- | --- |
| Low (≈ −1 SD) | 7.82 | 1.04 | −5.12 to 7.19 | 0.740 |
| Mean (W=0) | 18.69 | 5.47 | 1.17 to 9.77 | 0.013 |
| High (≈ +1 SD) | 29.56 | 9.91 | 3.73 to 16.08 | 0.002 |

Johnson–Neyman threshold (raw): 16.12 (centered value = −2.5697). Above 16.12, the effect is statistically significant (p < 0.05).

1. CMSS duration

| Moderator level | Baseline duration (raw) | Effect (Control − Intervention) | 95% CI | *p* |
| --- | --- | --- | --- | --- |
| Low (≈ −1 SD) | 8.76 | −0.65 | −6.72 to 5.43 | 0.833 |
| Mean (W≈0) | 20.00 | 5.55 | 1.31 to 9.80 | 0.011 |
| High (≈ +1 SD) | 31.00 | 11.62 | 5.70 to 17.55 | <0.001 |

Johnson–Neyman threshold (raw): 17.80 (centered value = −2.3312). Above 17.80, the effect is statistically significant (p < 0.05).

Conditional effects were estimated using PROCESS Model 1 with the baseline CMSS score mean-centered (W = baseline − mean).

Effects are reported as Control − Intervention; positive values indicate higher post-treatment scores in the control group (i.e., greater improvement in the intervention group).

Raw Johnson–Neyman thresholds were back-transformed as: threshold(raw) = threshold(centered) + mean(raw), using mean(raw) = 18.69090909 for severity and 20.12727273 for duration.

*p* values are two-sided; values <0.001 are reported as <0.001.

Supplementary Table S6. Sensitivity analysis of prostaglandin-related biomarkers excluding rescue ibuprofen users

| Biomarker | Intervention (n=26) Before | Intervention After | *Z* | *p* | Control (n=25) Before | Control After | *Z* | *p* | Post-treatment between-group *Z* | *p* |
| --- | --- | --- | --- | --- | --- | --- | --- | --- | --- | --- |
| PGE2 | 269.78 (168.60) | 406.78 (678.86) | -2.502 | 0.012 | 230.38 (116.44) | 277.02 (138.44) | -1.493 | 0.135 | -2.336 | 0.019 |
| PGF2α | 24.31 (15.67) | 17.01 (12.30) | -2.273 | 0.023 | 19.95 (13.26) | 20.17 (10.60) | 0.632 | 0.527 | -1.733 | 0.083 |
| PGF2α/PGE2 ratio | 0.08 (0.04) | 0.05 (0.06) | -2.832 | 0.005 | 0.08 (0.07) | 0.07 (0.05) | -0.040 | 0.968 | -3.165 | 0.002 |

Values are presented as median (IQR). Abbreviations: PGE2, prostaglandin E2; PGF2α, prostaglandin F2α; IQR, interquartile range.

Sensitivity analysis excluded participants who reported any rescue ibuprofen use during the treatment period.

Supplementary Table S7. Sex hormone levels (cycle days 2–7) at baseline and after three treated menstrual cycles

| Hormone | Intervention Baseline, median (IQR) | Intervention Post, median (IQR) | Control Baseline, median (IQR) | Control Post, median (IQR) | Group×Time *p*_raw | *p*_FDR (BH) |
| --- | --- | --- | --- | --- | --- | --- |
| LH (IU/L) | 6.15 (3.71) | 6.185 (4.05) | 5.58 (3.30) | 6.65 (3.56) | 0.871 | 0.871 |
| FSH (IU/L) | 6.10 (1.67) | 5.97 (2.20) | 5.75 (1.89) | 6.15 (1.79) | 0.067 | 0.134 |
| Estradiol, E2 (pmol/L) | 143.50 (101.13) | 168.00 (207.25) | 148.00 (96.00) | 163.50 (105.75) | 0.149 | 0.224 |
| Prolactin, PRL (mIU/L) | 362.00 (249.50) | 390.50 (199.25) | 320.00 (177.50) | 299.00 (164.00) | 0.413 | 0.496 |
| Progesterone, P (ng/mL) | 0.15 (0.16) | 0.215 (0.24) | 0.16 (0.12) | 0.16 (0.20) | 0.024 | 0.134 |
| Testosterone, T (nmol/L) | 0.66 (0.61) | 0.845 (0.63) | 0.76 (0.47) | 0.89 (0.46) | 0.046 | 0.134 |

Values are presented as median (IQR). Sex hormones (LH, FSH, estradiol, prolactin, progesterone, and testosterone) were measured on menstrual cycle days 2–7 at baseline and after three treated cycles. Group-by-time interactions were tested using exploratory linear mixed-effects models fitted on the ln scale with a random intercept for participants (fixed effects: Group, Time, and Group×Time). Multiplicity across six hormones was controlled using the Benjamini–Hochberg false discovery rate (BH-FDR; m=6). Testosterone is reported in nmol/L.

Supplementary Table S8. Rescue ibuprofen use patterns by menstrual cycle

Unit: Number of ibuprofen doses per menstrual cycle during the dysmenorrhea period (0 indicates no use)

A. Intervention group (n=55)

| Time point | Any use, n/N (%) | Doses per cycle, median (IQR) | 0 dose, n (%) | 1 dose, n (%) | 2 doses, n (%) | ≥3 doses, n (%) |
| --- | --- | --- | --- | --- | --- | --- |
| Baseline | 27/55 (49.1%) | 0 (0–1) | 28 (50.9%) | 14 (25.5%) | 8 (14.5%) | 5 (9.1%) |
| Cycle 1 | 24/55 (43.6%) | 0 (0–1) | 31 (56.4%) | 16 (29.1%) | 5 (9.1%) | 3 (5.5%) |
| Cycle 2 | 18/55 (32.7%) | 0 (0–1) | 37 (67.3%) | 11 (20.0%) | 3 (5.5%) | 4 (7.3%) |
| Cycle 3 | 19/55 (34.5%) | 0 (0–1) | 36 (65.5%) | 11 (20.0%) | 5 (9.1%) | 3 (5.5%) |

B. Control group (n=55)

| Time point | Any use, n/N (%) | Doses per cycle, median (IQR) | 0 dose, n (%) | 1 dose, n (%) | 2 doses, n (%) | ≥3 doses, n (%) |
| --- | --- | --- | --- | --- | --- | --- |
| Baseline | 29/55 (52.7%) | 1 (0–2) | 26 (47.3%) | 15 (27.3%) | 7 (12.7%) | 7 (12.7%) |
| Cycle 1 | 27/55 (49.1%) | 0 (0–1) | 28 (50.9%) | 14 (25.5%) | 7 (12.7%) | 6 (10.9%) |
| Cycle 2 | 27/55 (49.1%) | 0 (0–1) | 28 (50.9%) | 15 (27.3%) | 6 (10.9%) | 6 (10.9%) |
| Cycle 3 | 23/55 (41.8%) | 0 (0–1) | 32 (58.2%) | 12 (21.8%) | 6 (10.9%) | 5 (9.1%) |

Doses per cycle are summarized for all participants (including zeros). Percentages are calculated using the group total as the denominator (n = 55). “Baseline” refers to the cycle prior to randomization; Cycles 1–3 refer to the three treated cycles.
